# #Quarantineworkout: The Use of Digital Tools and Online Training Among Boxers and Boxing Coaches During the COVID-19 Pandemic

**DOI:** 10.3389/fspor.2020.589483

**Published:** 2020-11-20

**Authors:** Anne Tjønndal

**Affiliations:** Sport and Society Research Group, Faculty of Social Sciences, Nord University, Bodø, Norway

**Keywords:** digital technologies and sport, digitalization and digital transformation, digital tools and volunteer sport clubs, athletes and social media, COVID-19 and sports, digital qualitative research in sport, boxing and technology

## Abstract

The purpose of this article is to explore the use of online training strategies and digital tools amongst coaches and athletes in boxing clubs during the COVID-19 pandemic and the subsequent shutdown of organized sport. A digital qualitative research strategy was applied to boxing clubs, boxers, and boxing coaches in Norway. A total of 46 official clubs, athletes, and coach pages on Facebook were followed from 12th March to 30th June 2020, resulting in a sample of 78 social media posts (texts, photos, and videos). A content analysis approach was used for the material. The results show that the use of digital tools amongst the studied Norwegian coaches and boxing clubs varied in frequency and form during the spring of 2020 (COVID-19 shutdown). For them, the most frequent use of digital instruments was to communicate internally and externally about the COVID-19 situation, national rules and guidelines. The material demonstrated that online training strategies varied between different constellations of three specific factors: (1) synchronized (live-streamed) online training and unsynchronized online training (at home training videos and programmes), (2) publicly published online training that was only accessible through digital registration, and (3) free online training and online training that was only available to paying members. For the athletes in the material, the most frequent content was social media posts for self-promotion purposes. Additionally, several of the athletes expressed that they struggled to cope with and manage the training at home during lockdown, and that they deeply missed training and competing as usual.

## Introduction

The COVID-19 pandemic of 2020 is the first time since World War II that organized sport has ground to a halt and athletes have been unable to compete internationally (Pedersen et al., 2020). In the spring of 2020, elite and grassroots sports were shut down around the world in order to contain the coronavirus and prevent it spreading. Global sports mega events, such as the Tokyo 2020 Summer Olympic Games, were cancelled and postponed until 2021 (IOC, 2020a). In the case of boxing, the 2020 AIBA Youth Women's and Men's World Championships and multiple annual national and transnational championships, such as the England Boxing National Amateur Championships and the Nordic Boxing Championships (AIBA, 2020), were cancelled. The qualifying events for Tokyo 2020 have been postponed to 2021 (IOC, 2020b), and a European qualifier tournament that was scheduled to run from March 14 to 24 was cancelled after only 2 days of competition, leaving the slots for the Olympic Games in 2021 unfilled.

Due to the pandemic, sports that were once part of daily life for athletes around the world have been cancelled for an indefinite period. After several months of cancelled competitions, closed sporting facilities, national government restrictions, and social distancing requirements, regions have now slowly begun to reopen sports facilities and allow some organized sports to re-engage in their core activities. However, organized sport is still a long way from being “back to business” as usual, and sports like boxing—that cannot be practised without extensive full-body contact—are subject to the harshest restrictions for the simple reason that it is not possible to practise full-contact sports competitively and maintain the required social distance. In other words, while organized sports in general have been seriously affected by the COVID-19 pandemic, boxing and other full-contact sports are likely to experience longer periods of shutdown and more severe restrictions than non-contact sports.

As the time of writing this article COVID-19 is an ongoing pandemic. The consequences of the pandemic and the subsequent shutdown of sport in society are still uncertain. Some research in the field of sports medicine has also raised concerns about the impact of the coronavirus disease on athletes' health. For instance, Baggish et al. (2020) argue that due to the restrictions on training and competition, the resumption of competitive sport will mean challenges in ensuring the cardiac safety of the millions of athletes worldwide. Similarly, Paoli and Musumeci (2020) highlight that long-term detraining, as in this forced COVID-19 stop, leads to a decline in maximal oxygen consumption (VO2max), a loss of endurance capacity and a loss of muscle strength and mass. According to Paoli and Musumeci (2020), these factors will significantly increase the risk of injury amongst elite athletes. Although these concerns are valid, little research has been done on how the shutdown of sport during the COVID-19 pandemic has affected the training habits of athletes in different sports and different socio-geographical locations. Have athletes simply ceased to train and engage in sport during the lockdown? Or have they adapted to the situation and developed new training strategies? More specifically, how has the pandemic impacted the use of digital tools and digitalization in sport? How do athletes use digital training strategies, such as participation in online training, during lockdown? This article aims to shed light on these issues by analysing the use of digital tools in Olympic boxing during the COVID-19 lockdown.

Specifically, the research question guiding this study is: *How have boxers, boxing coaches, and boxing clubs made use of digital tools and online training strategies during the COVID-19 lockdown?* This question is explored by means of a qualitative analysis of social media content (Facebook) on the topic of the COVID-19 shutdown of organized sport in Norway and novel digital training strategies (pictures, videos, and texts) published by Norwegian boxers, boxing coaches, and boxing clubs. Like most social sports-related phenomena this research question could easily be explored through a multitude of methodological approaches, such as qualitative interviews or a quantitative survey. Social media content analysis was chosen as a strategy for this study mainly because during the COVID-19 lockdown, Norwegian boxing clubs began, for the first time, to promote online training activities. In most cases, the use of such novel digital tools was announced on the social media accounts of clubs and coaches. Since some coaches and clubs advertised open online training free for anyone to join, this content appeared as unique data to gain insight into the use of digital tools among boxers, boxing coaches, and boxing clubs during the COVID-19 pandemic. On an initial screening of the most common social media platforms used by athletes and coaches (Twitter, Facebook, Instagram, Tik Tok, Snapchat), Facebook stood out as the platform with the most diverse public (official) accounts. That is, boxers, boxing coaches, and boxing clubs were all represented on Facebook. On the other social media platforms, the same athletes (as on Facebook) could be identified but fewer coaches and club accounts were available for analysis.

In the following, I present a brief summary of previous research on digitalization and the use of digital tools in organized sport. This is followed by a description of my material and methodological approach. Thirdly, the results are presented and discussed in relation to findings from previous studies about the use of digital tools in sport.

## Analytical Framing

In order to examine the use of digital tools and online training strategies amongst boxers, boxing coaches, and boxing clubs in Norway during the COVID-19 lockdown, I combine previous research on digital tools and digitalization in sport with the theoretical concepts of path dependence/path disruption and critical junctures in an attempt to understand how individuals, groups, and organizations respond to unexpected change (exogenous shocks) and the potentially lasting consequences of critical external events.

### Technology, Digitalization and the Use of Digital Tools in Sport

Digitalization has become, and continues to be, a vital component in all realms of life, of which sport is no exception. Throughout the years, sports organizations have seen advances in fields such as sports medicine and injury prevention (Ventresca, 2019; Rigamonti et al., 2020), performance monitoring and measurement (Miah, 2017; Johnson, 2020), fan interaction and communication (Hinck, 2019), and sports equipment (Balmer et al., 2012). Digital tools increasingly shape workflows in sports organizations (Torres-Ronda and Schelling, 2017) and provide a wide range of applications for organizational development, including administration, internal and external communication, administration and improved control of training and competition processes (Ehnold et al., 2020). For instance, coaches and athletes communicate via digital platforms and video-conferencing, grassroots sports clubs advertise their activities on social media, and high performance sports teams make decisions about how to develop their brands further based on computer-generated algorithms. Some scholars have illustrated how digital technologies create novel challenges for sports organizations. These include online bullying and virtual maltreatment of athletes (Kavanagh et al., 2019), sexism toward female sports fans (Radmann and Hedenborg, 2019), and social inequality of access to digital tools (Tjønndal, 2021). Despite the deep impact of digital technologies on sport, research on the use of digital tools in organized sports is still rare and dispersed.

Much of the literature on digital tools and digitalization has focused on professional sports clubs and analysed the implementation and application of digital technologies in high performance sport. Hence, the use of digital technologies for opening up new target groups and marketing opportunities has been examined in some detail, including sports fans use of mobile apps and online platforms (Kang, 2015; McGillivray and McLaughlin, 2019; Qian et al., 2019), elite athletes use of social media platforms (Geurin-Eagleman and Burch, 2015; Chawansky, 2016), resistance to digitalization in elite sport (Trabal, 2008; Tjønndal, 2020), social media activities and corporate communication (Waters et al., 2010; Yan et al., 2018) as part of sports clubs' branding strategies (Watkins and Lewis, 2014; Bertschy et al., 2019), and online educational and administrative resources for sports organizations (Sellitto et al., 2016; Strachan et al., 2016). A notable exception to the literature on technology and digitalization in professional sports is Ehnold et al., 2020 study of voluntary sports clubs and their use of digital tools. Ehnold et al. (2020) conducted an online survey of voluntary sports clubs in Austria and Germany (*n* = 787) with the aim of identifying and describing their digital use behaviour. The statistical analyses of Ehnold et al. (2020) showed that 93.7% of the surveyed sports clubs reported using digital instruments for internal and external communication. The second most reported use of digital tools was “to report membership data to federations” (82.1%). Their findings thus indicate that at present digital instruments in voluntary sports clubs are primarily used for internal/external communication and conventional administrative tasks. Furthermore, Ehnold et al. (2020) identified goals of success in competitive sports and cooperation with other institutions as two promoting factors for how voluntary sports clubs use digital tools. Additionally, the sports clubs with a high proportion of volunteers with administrative tasks used digital tools more frequently. However, according to Ehnold et al. (2020), the strongest restriction on the use of digital instruments can be observed in voluntary sports clubs that report that “digital processes do not fit with club culture” and when the organization does “not have a clear strategy for the digitalization of our club.”

While Ehnold et al. (2020) do not discuss specific digital tools [such as smartphone applications (apps), online forums, or social media platforms], some studies have explored the use of certain digital tools in sports organizations. Rigamonti et al. (2020) argue that the ever-increasing number of apps used in sport and fitness contexts are marketed to a diverse audience, which means that the myriad of apps that are available provide useful information for health conscious individuals (Higgins, 2016) and dedicated professional athletes (Peart et al., 2019) alike. Specifically, Rigamonti et al. (2020) illustrate how app-based diagnostics solutions have the potential to improve concussion screening in elite sport. Similarly, van Tuyckom (2021) examines how the development of an app, as a co-creation between participants, public sector stakeholders, and voluntary sports clubs, can support prolonged sport participation among socially vulnerable youth in Bruges, Belgium.

Some critical studies of increased digitalization and the use of digital tools in sport have been carried out. Peart et al. (2019) argue that the validity and reliability of the performance and health data collected through sport and fitness apps is often unknown. Peart et al. (2019) acknowledge the potential of mobile apps to collect data in the sports field, but advise athletes and practitioners to exercise caution when using them because not all apps are developed based on research. In another critical study of smartphone apps, Rist and Pearce (2016) tested the hypothesis that apps could improve athletes' engagement in mental training programmes. To test this hypothesis, they recruited 46 male adult athletes in professional Australian Rules football to participate in their study. The players were randomized into three groups to use one of three apps over a 4 week period. Their results showed that player engagement was noticeably reduced in all three groups with compliance falling, compared to initial participation levels (before using the apps). Hence, they concluded that smartphone apps do not improve compliance with mental training programmes or significantly improve outcomes among athletes.

Finally, some research suggests that social and demographic factors, such as gender, social class, and ethnicity, affect the use of health- and sports-related wearable digital technologies. In a study of undergraduate students use of wearable devices, Pan et al. (2019) found that men were more likely than women to use wearable technology. Similarly, in a study of gender and digital games, Crawford (2005) found that women were a lot less likely than their male peers to play digital games, and when they did played them less frequently. Pan et al. (2019), on the other hand, found no significant relations between wearable device use and social class standing or academic status. In contrast to the findings of Pan et al. (2019), the findings of a study of Australian youth indicated that there was a strong link between technology use and social class (North et al., 2008). Although the youths had similar access to digital technologies at home and at school, and similar knowledge of these technologies, their practices varied according to their social background. The youths whose parents were highly educated and were in high status occupations used technology more frequently and diversely than other youths (North et al., 2008).

### Path Dependence and Critical Junctures

In the literature on path dependence, there is a general notion that previous occurrences will shape how an individual, group, or organization develops over time. One of the classic definitions of the term was proposed by Sewell in 1996, where path dependence is described as events where “*what happened at an earlier point in time will affect the possible outcome of a sequence of events occurring at a later point in time*” (1996: 262–263). However, Nilssen (2019a) 2019b argues that as Sewell (1996) definition is relatively open, the concept of path dependence is sometimes used in an unnuanced way, where perspectives on path dependence are so vague that they boil down to arguments along the lines of “history is of significance” (Nilssen, 2019a). Nevertheless, in general, theories of path dependence do argue for the importance of timing and sequence, departing from similar conditions, many possible outcomes, and extensive impact from relatively minor and (seemingly) insignificant events at a later date (Pierson, 2000).

A common perception of path dependence is that the impact of an early course of action will be as good as irreversible, i.e., that early path-shaping choices can lead to critical moments in social, organizational or political development (Collier and Collier, 2002). Pierson (2000) emphasizes that it is not only large-scale events or decisions that can have an extensive impact on groups of people or organizations, but also that seemingly small and contingent events can affect them if the timing is right. Specifically, Pierson (2000) outlines a list of four key features that define path-dependent (or path-disruptive) processes: (1) many possible outcomes (a wide range of outcomes are possible), (2) contingent context (small events can have an extensive and lasting impact if they happen at the right time), (3) timing and sequencing (when an event occurs is significant), and (4) organizational delay (when a process has been initiated, positive resolution centred on one specific option can result in this option being dominant, and, consequently, the one chosen. The selected option will then later be resistant to change).

Pierson's list of defining features of path dependence/path disruption shares several features with Collier and Collier (2002) framework of critical junctures. Here, a critical juncture is defined as “*a period of significant change, which typically occurs in distinct ways in different countries (or in other units of analysis) and which is hypothesized to produce distinct legacies*” (Collier and Collier, 2002: 29). Similar to the theory of path dependence, the framework of critical junctures also explains why some events or decisions have a lasting impact (if they do not have a lasting impact, they are not critical). These theories share a common feature—they both emphasize timing and context as important for the result, so that when the critical juncture occurs, the consequences will be significant and difficult to reverse.

Critical junctures are understood as incidents that are beyond the control of the individual, organization or local government, with the potential to notably influence and change practices (Salamonsen, 2015). In other words, critical junctures can act as catalysts for path disruption. Commonly, critical junctures are viewed as occurrences caused by exogenous factors (Nilssen, 2019a). In other words, critical junctures are unexpected events that catalyse an unpredictable window for action and/or change.

## Materials and Methods

This article is based on an inductive approach to qualitative inquiry. Methodologically, the data derives from a digital research strategy. According to Bundon (2016:356), digital research in the sports sciences can be described as research strategies that “*include the use of digital tools to collect data and the collection of digital data*.” Bundon (2017) also highlights that digital qualitative research methods include explorations of public and private cyberspaces, researcher participation and non-participation in online spaces, as well as analyses of authored published work and anonymous online data. Following Bundon (2016) 2017 methodological approaches, the exploration of the use of digital tools and online training strategies by boxers, boxing clubs, and boxing coaches presented in this article is based on data from public cyberspaces, with non-participation from the researcher in the online spaces, and analyses of authored published works.

### Sample

The data consists of Facebook posts of Norwegian boxers, boxing coaches, and boxing clubs published between 12th March and 30th June 2020. 12th March was set as the earliest publication date for social media content included in the sample because this was when the Norwegian Boxing Federation (NBF) officially shut down all national boxing activities due to the COVID-19 pandemic (NBF, 2020).

**Figure 1:**
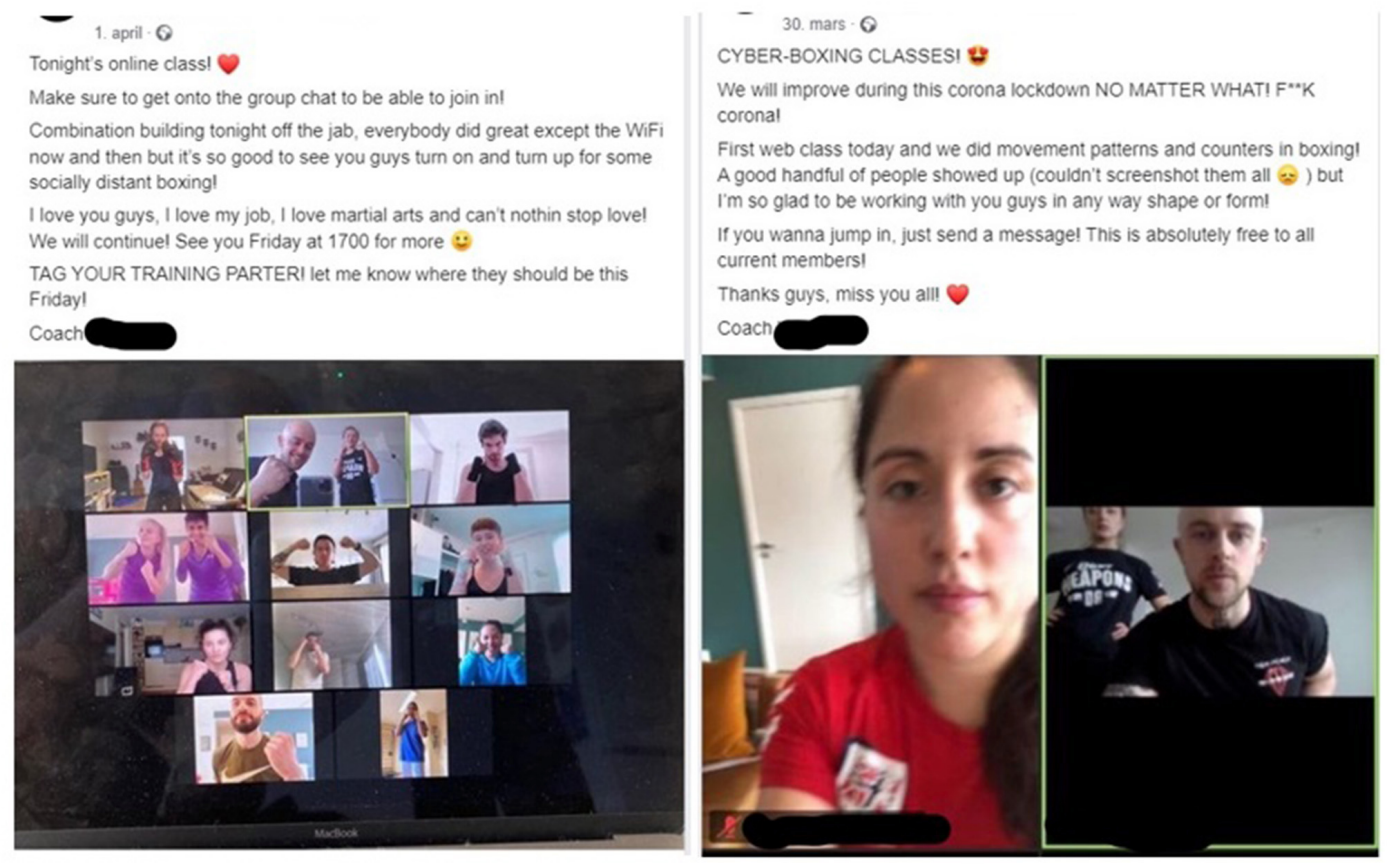
Examples of synchronized (live streamed) online boxing classes during COVID-19. Retrieved from: https://www.facebook.com./thenorwegiancombatacademy/posts/637173153511946 and https://www.facebook.com./ithenorwegiancombatacademy/posts/635991320296796.

**Figure 2:**
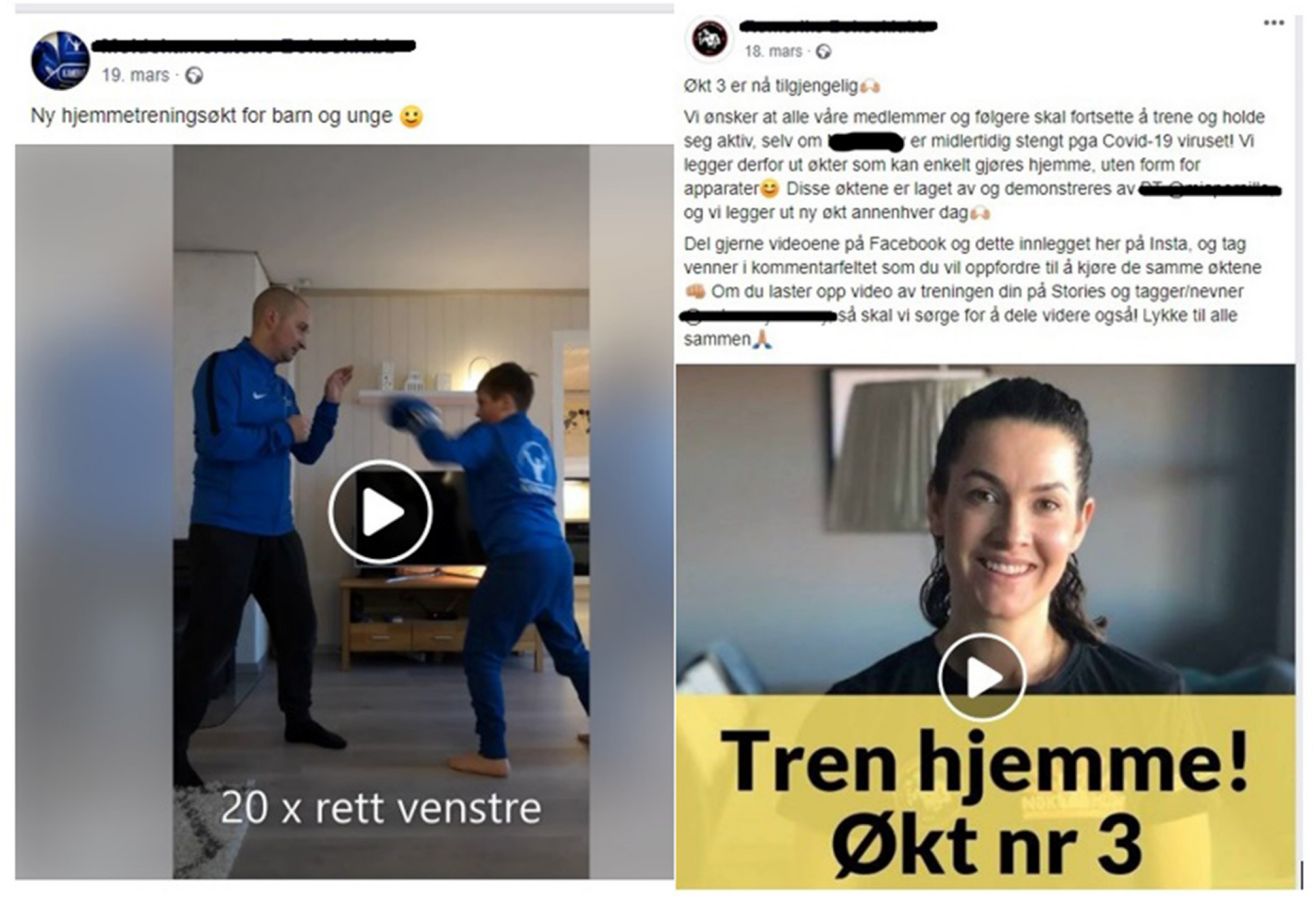
Examples of unsynchronized online boxing classes during COVID-19. Retrieved from: https://www.facebook.com./romerikebokseklubb/posts/2528531147463732 and https://www.facebook.com./moldekameratene/posts/2527852790768664.

**Figure 3:**
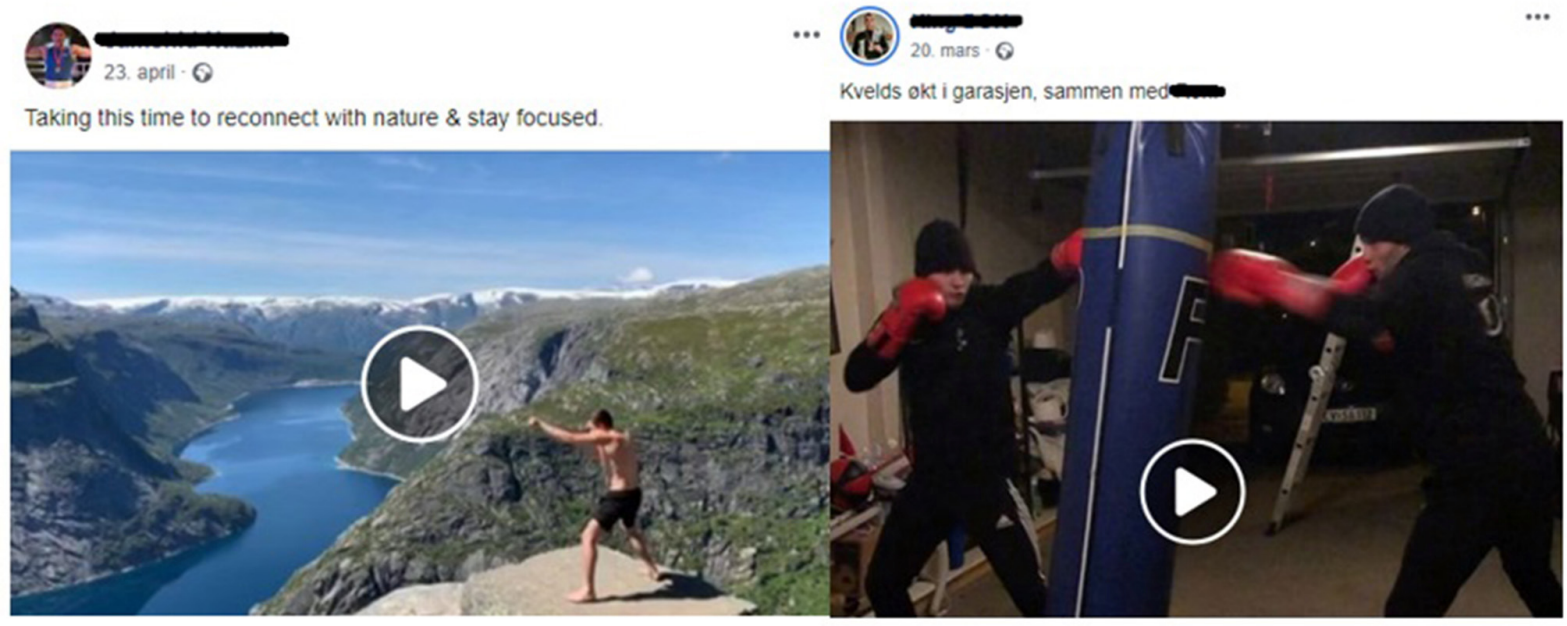
Examples of self-promotion and training motivation content. Retrieved from: https://www.facebook.com.permalink.php?storyfbid=2959047314157443&id=2057925467602970 and https://www.facebook.com./TeamNazari/posts/3528395930510551.

To determine the sample, I followed all the official Facebook pages of Norwegian boxing clubs, coaches, and boxers registered with NBF during the COVID-19 lockdown of boxing in the spring of 2020 (as indicated above, from 12th March to 30th June). I only included social media posts relating to public and official athletes, coaches, and club pages in my material. While Facebook was the selected social media platform for the data collection, many athletes and clubs re-posted material from other social media accounts (such as Instagram and TikTok), or linked to content on other platforms, such as YouTube, Zoom, or blogs. The search for material was limited to posts that included the topics online training and the use of digital tools to manage the COVID-19 lockdown. This strategy resulted in a sample of 78 social media posts from Norwegian boxers, boxing coaches, and clubs for analysis (see Appendix I for a complete list of the data material). In accordance with national ethical guidelines for internet research (NESH, 2019), in the cases in which I present photos (screenshots) from the material (see Figures 1–3), verbal and written consent was collected from the athletes and coaches concerned. The names of the boxing clubs, coaches, and athletes in the photos presented in Figures 1–3 have been removed to protect the participants' identities at much as possible. They could not be anonymized completely, though, in that the figures contain photos of individuals.

The sample consists of different types of texts. The main body of texts in the sample are Facebook updates written by the athletes and coaches themselves. Many of these include photos, videos, and links to other social media accounts. An overview of the sample with links to each social media post is provided in Appendix I. At the time of the data collection, all the social media posts (including videos) were publicly available online.

### Analysis

The material was analysed using a qualitative content analysis approach (Vaismoradi et al., 2013). Content analysis uses a descriptive approach in both the coding and interpretation of the data. When analysing, categorizing and coding the material, my aim was to describe the characteristics and content of each social media post (texts, photos, videos) by examining “who says what, to whom and with what effect” (Bloor and Wood, 2006). Using this approach, the analysis of the material resulted in three distinct themes, which are highlighted in the results section of the article: (1) online training strategies to manage the COVID-19 pandemic shutdown, (2) digital tools as a means of communication and administration, and (3) self-promotion, awareness, and motivational content. Most of the texts included in the study were originally published in Norwegian. The description of the texts and the quotations from them have been translated from Norwegian to English by the author.

As the coding and analysis of the data was done solely by the author, this part of the research process raises some validity issues. As Eggebø (2020) argues, when multiple researchers engage in qualitative analysis collectively, it enables a more creative analytical process and strengthens the validity of the presented findings. Multiple researchers working collectively with qualitative analysis might strengthen validity as Eggebø (2020) suggests, although I would argue that this is only true to a certain extent. There are limitations to how collective analysis can strengthen analytical validity. Notwithstanding, doing qualitative analysis as an individual endeavour, as I have done in this study, is potentially a limitation of the analytical process. Moreover, it is not the goal of this study to generate the best possible validity, but to generate new insights into a novel research field—the use of digital tools by boxers, boxing coaches, and clubs during the COVID-19 pandemic of 2020.

## Results

A total of 46 official (public) Norwegian boxing clubs', coaches', and athletes' Facebook pages were followed daily during the period 12th March to 30th June 2020. During this period, 23 of the 46 Facebook pages posted content relating to COVID-19, online training, and the use of digital tools. From these 23 Facebook pages, a total of 78 social media posts were identified, consisting of texts, photos, and video material. The analysis of the content in these posts is presented below, organized according to the three topics identified in the content analysis: (1) online training strategies, (2) self-promotion, awareness, and motivational content, and (3) digital tools, communication, and administration.

### Online Training Strategies

Only two of the athletes' Facebook pages analysed in the material posted content relating to online training strategies during the COVID-19 lockdown (see Bernard Angelo Torres and Alexander Hagen in Appendix I), while several of the boxing clubs' and coaches' pages posted content on the topic of online training. In the material, there are notable differences in strategies between the boxing clubs and coaches when it comes to online training. These differences relate to three different factors: (1) synchronized (live-streamed) online training and unsynchronized online training (via home training videos and programmes), (2) publicly published versus accessible training through registration, and (3) free online training and training sessions available only to paying members.

A common strategy amongst the boxing clubs and coaches was synchronized (live-streamed) boxing trainings using digital platforms such as Zoom and Microsoft Teams (for empirical examples, see for instance The Norwegian Combat Academy and Bodø Bokseklubb in Appendix I). Two examples of synchronized online training found in the material are presented in Figure 1.

While some boxing clubs (Figure 1) arranged online synchronized training exclusively for members of the specific club, others used Facebook to post links to live-streamed training sessions that were open to anyone interested in participating (for empirical examples in the material see Bodø Bokseklubb in Appendix I). The boxing clubs and coaches that limited synchronized online training sessions to paying members/athletes via digital sign-up services argued that this was a necessary safety precaution (see Oslo Bokseklubb in Appendix I for example). On the other hand, boxing clubs and coaches that chose to make their synchronized trainings publicly available promoted this strategy as a way of recruiting new members to the club when the COVID-19 lockdown was over (see for instance Romerike Bokseklubb in Appendix I). As Bodø Bokseklubb stated in its Facebook event for digital boxing training: “This is an open access boxing training session available for anyone who would like to train boxing and condition at home during the COVID-19 lockdown. The digital training session is free and you do not have to be a member of Bodø Bokseklubb to participate” (see Bodø Bokseklubb in Appendix I).

Some boxing clubs and coaches chose to post unsynchronized online training videos for home boxing and condition training during the COVID-19 shutdown of organized sport (see Figure 2, and Moldekameratene Bokseklubb and Romerike Bokseklubb in Appendix I). In the material, these videos feature technical drills and condition exercises that parents can do together with their children (youth boxers), and conditioning exercises that do not require any sport/exercise equipment (Figure 2). Some boxing clubs posted new training sessions and videos as often as every other day. One of the boxing clubs described this online training strategy in this way: “*We want all our members and followers to keep training, even if we are temporarily closed because of the COVID-19 virus! Therefore, we will be posting training sessions that you can do at home without any equipment*” (translation from Figure 2).

### Coping With Lockdown: Self-Promotion, Awareness and Motivational Content

Among the boxers who posted COVID-19 related content on their Facebook pages during the lockdown of Norwegian boxing, the most common were self-promotional and motivational photos, videos, and texts. Many of the boxers in the material posted photos and videos from their “*#quarantineworkout*” in their homes, or outside in their neighbourhoods (Figure 3), with motivational quotes such as “*Even if we have to stay at home these days, we can still do workout. There's lots of training we can do inside our home #stayhome #socialdistancing #COVID19*” (see Bernard Angelo Torres in Appendix I). Additionally, several boxers posted content expressing their struggles with not being able to go to the boxing gym, and that they missed training and competing. There are many examples of this in the material, with statements such as “*I'm making the best of these trying times*” (see Anniken Holthe Boxing in Appendix I) and “*I can't wait to be back in the ring*” (see Bernard Angelo Torres in Appendix I). Some of the boxers' Facebook pages also contained posts with content that was meant to raise awareness of the COVID-19 virus, encouraged people to “*Follow national guidelines, take care of yourself and your loved ones*” (see Simen Nysæther in Appendix I) and reminded fellow boxers that “*health and safety always comes first*” and to “*stay safe*” (see Anniken Holthe Boxing in Appendix I).

Motivational content was also common amongst the boxing clubs and coaches in the material (see for instance Jessheim Bokseklubb in Appendix I). In particular, many clubs posted videos of members doing boxing workouts at home, applauding their efforts with captions like “*Our coaches and athletes are getting COVID-19 creative*!,” “*The boxing gym has been swapped with garages, basements, living rooms, neighbourhoods, and forests, but we endure!*,” “*Only the imagination can stop our amazing athletes*,” “*We are boxers. We adapt and overcome!*.”

While many athletes posted content expressing how much they missed boxing, some boxing clubs and coaches posted content that raised awareness of their club's condition during the lockdown, and the need for members to keep paying their membership fees. One of the coaches in the material expressed it in this way:

This is a scary time. I thank you all from the bottom of my heart for keeping your payments running, I know it is a hard time and I am of course working closely with those who have hard economic times but the gesture of continuing your payments is literally the ONLY thing keeping the boxing gym in business. This gym is the result of over 14 years, half a life of work and it means the world to me, it IS me! So I cannot thank you enough for keeping this new and vulnerable gym open! Next week we will be taking online classes on ZOOM on Monday, Wednesday and Friday at 17.00 and will run for 40 minutes. We've gotta stay active and we've gotta stay sharp!

One of the clubs went as far as giving away “corona gifts,” consisting of skipping ropes and other boxing exercise equipment that could be used at home, to boxers who continued to pay their membership fees during the shutdown (see Moldekameratene Bokseklubb in Appendix I).

### Digital Tools, Communication, and Administration

The boxing clubs' and coaches' most frequent use of digital tools was Facebook and social media as a platform for communication and administration. All the boxing clubs and coaches in the analysed material (Appendix I) used digital tools for communication and/or administration purposes during the COVID-19 lockdown in the spring of 2020. Most commonly, clubs and coaches used social media to repost the guidelines and rules from NBF and the Norwegian Olympic and Paralympic Committee and Confederation of Sports (NIF) on the COVID-19 pandemic.

When boxing clubs were allowed to host socially distanced (2 m distance between participants) training sessions outdoors for a limited number of athletes (at first in groups of 5 and later in groups of 20), many boxing clubs and coaches started using digital tools to administer boxers signing up for training sessions to make sure they adhered to the national COVID-19 guidelines (see for instance Oslo Bokseklubb in Appendix I).

## Discussion and Conclusion

First of all, it is clear that the use of digital tools by boxers, boxing coaches, and clubs has varied greatly during the COVID-19 lockdown in the spring of 2020. Some clubs and coaches only posted 1–2 updates with information that the boxing gym would be closed until further notice, while others hosted live-streamed training sessions every other day for months. Hence, the use of digital tools and online training strategies has varied both in frequency and form.

All the boxing clubs in the analysed material used digital tools for internal and external communications, as well as for administrative tasks related to the management of members in accordance with national COVID-19 guidelines for organized sport. These findings are consistent with previous findings on the use of digital tools in voluntary sports clubs in Austria and Germany (Ehnold et al., 2020). In their studies, Ehnold et al. (2020) found that sports clubs with a high proportion of volunteers with administrative tasks used digital tools more frequently. This may explain why some boxing clubs made use of online training strategies and others only used digital tools for necessary communications during the COVID-19 lockdown. However, the currently available data does not enable me to determine whether this is the case here. That that some boxing clubs and coaches only used digital tools for necessary communications (e.g., to post that the boxing gym was closed due to the COVID-19 pandemic) could also be interpreted as a form of resistance to digitalization in sport, as explored in a few previous studies (Trabal, 2008; Sellitto et al., 2016; Tjønndal, 2020). As Trabal (2008) found, coaches are more likely to resist technological innovation than athletes. Sellitto et al. (2016) argue that many sports organizations do not use digital tools because they do not have the expertise to exploit them productively. This may also be the case for the boxing clubs and coaches making limited use of digital tools and online training strategies during the shutdown.

The boxers included in the material mainly used digital tools for self-promotion during the COVID-19 pandemic shutdown. This finding is consistent with those in the studies undertaken by Geurin-Eagleman and Burch (2015) and Chawansky (2016). Specifically, Geurin-Eagleman and Burch (2015) show that male and female athletes use social media to build their personal brand and for brand management. This is also the case here, as many athletes posted content thanking their sponsors for supporting them during the COVID-19 crisis (see for instance Camilla Johansen, Mindaugas Gedminas and Bernard Angelo Torres in Appendix I). However, Geurin-Eagleman and Burch (2015) also found that female athletes were more likely to share photos of themselves than their male counterparts, and that the photos were mainly taken in private settings. This is not the case in this study, though, as most boxers (both male and female) posted photos and videos of their “#stayhome #quarantineworkout” from home. It may be that these findings diverge from that of Geurin-Eagleman and Burch (2015) due to the lockdown situation. Pan et al. (2019) found that in their sample, men were more likely to use digital technologies than women. In my sample there are also more men than women. However, the majority of the sample consists of posts from boxing clubs and coaches. Furthermore, boxing is a male dominated sport (Rana, 2017; Tjønndal, 2019), which means there more males than females are involved. Hence, the data that is analysed here is limited in terms of examining how gender influenced the use of digital tools in sport during the COVID-19 pandemic. This is also the case for influences of other social and demographic factors, such as income, academic background, and social class. Pan et al. (2019) found no link between social class and/or academic background in the use of digital technologies. North et al. (2008), on the other hand, concluded that social class strongly affects how youth use digital technologies. As technology is embedded in relations of power and serves the interest of individuals or institutions, whether for economic or cultural means, it is likely that factors like gender and social class influenced athletes use of digital tools and online training strategies during the pandemic lockdown. In order to examine such differences, more research is needed on this topic. In this context, quantitative surveys and qualitative interviews would be more suited to uncovering potential differences rather than the social media data analysed for this article.

The material demonstrates that many boxing clubs and coaches have increased their use of digital tools during the COVID-19 lockdown. In particular, many clubs have incorporated online training strategies, both live-streamed and pre-recorded videos, which were not offered prior to the national shutdown of organized sport in Norway. This is perhaps not very surprising, given that COVID-19 represents an exogenous shock (or critical juncture) (Collier and Collier, 2002; Nilssen, 2019a 2019b) for athletes, coaches, and clubs. In other words, the pandemic and the subsequent lockdown of society represents a period of significant change and an event that demands action and adaptive skills from athletes, coaches, and sports clubs. From this theoretical perspective (Sewell, 1996; Pierson, 2000), clubs, coaches, and athletes have their set ways of organizing sports practices and competitions (path dependence).

Collier and Collier (2002) state that a feature of critical junctures is that they are often hypothesized to produce distinct legacies, which means that critical junctures can potentially lead to path disruption (i.e., lasting change) (Nilssen, 2019a). However, it remains unclear as to whether the COVID-19 lockdown will contribute to a lasting change in terms of training habits amongst athletes, novel ways of organizing sports activities and competitions for boxing coaches and clubs, or an increased digitalization of Norwegian boxing clubs. The available data does not enable me to answer such questions, but future research could evaluate the lasting impacts of the COVID-19 pandemic. If boxing clubs and coaches abandon online training strategies once the lockdown is over, and boxing training and competitions resume as normal, it would indicate that even though the pandemic can be characterized as a critical juncture (or exogenous shock), individual and organizational practices in boxing would still best be described as path dependent following the pandemic (Sewell, 1996; Pierson, 2000). In this case, COVID-19 as a critical juncture would not lead to path disruption in boxing practices in Norway. Just as the COVID-19 crisis has forced some clubs and coaches to use digital tools, it is likely that this exogenous shock will in fact lead to path disruption among boxers, boxing coaches, and boxing clubs. According to the previous findings of Ehnold et al. (2020), a likely outcome would be an increase in the use of digital tools by boxing clubs and coaches for the registration and administration of members post COVID-19. However, this remains to be seen, and follow-up studies are needed to explore whether or not the shutdown has contributed to an increased use of digital tools by boxing clubs and coaches.

In the light of the theoretical concepts of path dependence, path disruption and critical junctures (exogenous shocks) (Pierson, 2000; Collier and Collier, 2002), the increased and novel use of online training strategies by Norwegian boxing clubs and coaches during the COVID-19 lockdown can be interpreted and analysed as cases of crisis management in voluntary sports clubs. Studies of innovation in public governance have illustrated that a crisis situation may often inspire innovation in the organization (Nilssen, 2019a,b). It may also be the case for Norwegian boxing clubs and coaches that the COVID-19 pandemic has inspired digital innovation in communication, administration and the organization of training and core activities. It remains to be seen whether these novel uses of digital tools will continue past the COVID-19 pandemic, and will in turn transform the organizational culture of voluntary sports clubs, such as the boxing clubs studied here.

### Limitations

The present study should be read as an early contribution to the limited research topic of digitalization and the use of digital tools in voluntary sports clubs (Ehnold et al., 2020). However, the study has a number of limitations and unexplored perspectives that need to be taken into consideration in further investigations. These are: (1) As there are several validity issues with internet research, a broader data base (both quantitative and qualitative) from other sporting contexts is needed to validate the results and test them for methodological errors such as sample biases. Further research on the use of digital tools and online training in voluntary sports clubs during the COVID-19 pandemic would help to expand the scope of knowledge. (2) Social media data is just one of several methodological approaches that could be applied to study how boxers, boxing coaches, and boxing clubs use digital tools and online training strategies during a pandemic. Quantitative surveys and qualitative interview data could provide other insights into this phenomenon, and perhaps be better suited to examining how factors such as gender, age, academic background/social class influence the use of digital tools in a crisis situation. (3) The social media platform Facebook was utilized to identify the sample. Previous research has indicated that Facebook might not be the best platform to investigate the use of digital tools amongst athletes and coaches. In their study of elite sports clubs' branding strategies, Watkins and Lewis (2014) found that Facebook was a less popular social media platform than Twitter. Similarly, Chawansky (2016) states that Instagram now has more users than Twitter. Further studies of the content in other platforms may be more appropriate and yield novel insights. (4) The COVID-19 pandemic represents a global crisis that has forced athletes and coaches to manage an unknown situation. The extreme circumstances of the lockdown of organized sport is perhaps an interesting case for the study of the use of digital tools in sport, although on the other hand, it does not represent the everyday life of sports organizations. Hence, it is uncertain as to whether the data analysed here is comparable with findings from previous studies of digitalization and the use of digital tools amongst athletes, coaches, and voluntary sports clubs.

## Data Availability Statement

The original contributions presented in the study are included in the article/Supplementary Materials, further inquiries can be directed to the corresponding author.

## Ethics Statement

Informed consent was obtained from the sports clubs and individual(s) for the publication of any potentially identifiable images or data included in this article.

## Author Contributions

The author confirms being the sole contributor of this work and has approved it for publication.

## Conflict of Interest

The author declares that the research was conducted in the absence of any commercial or financial relationships that could be construed as a potential conflict of interest.
