# Supplementary material for: #Quarantineworkout: The Use of Digital Tools and Online Training Among Boxers and Boxing Coaches During the COVID-19 Pandemic
Source: Front Sports Act Living. 2020 Nov 20;2:589483. doi: 10.3389/fspor.2020.589483 (PMC7739676; doi:10.3389/fspor.2020.589483)
Supplement: Supplementary file 1 [file Data_Sheet_1.DOCX]

**Appendix I: Data material**

Alexander Hagen (March 12th 2020). In Facebook [Official athlete page]. Retrieved

29.07.2020 from: <https://www.facebook.com/norwayboxpromotion/photos/a.114448023291906/224945958908778/?type=3>

Alexander Hagen (June 4^th^ 2020). In YouTube [Official athlete account]. Retrieved

29.07.2020 from: <https://www.youtube.com/watch?v=izzRDS_CbLs&feature=youtu.be&fbclid=IwAR2j06da3f0KhlC5FJuPNH1KVAHDxOX4VlxjG520-zQqCGtGSadWqclt7Xw>

Anniken Holthe Boxing (March 22nd 2020). In Facebook [Official athlete page]. Retrieved

29.07.2020 from: <https://www.facebook.com/AnnikenHolthe/posts/154300456043322>

Anniken Holthe Boxing (March 29th 2020). In Facebook [Official athlete page]. Retrieved

29.07.2020 from: <https://www.facebook.com/AnnikenHolthe/posts/156642205809147>

Anniken Holthe Boxing (April 4th 2020). In Facebook [Official athlete page]. Retrieved

29.07.2020 from: <https://www.facebook.com/AnnikenHolthe/posts/164039981736036>

Anniken Holthe Boxing (April 10th 2020). In Facebook [Official athlete page]. Retrieved

29.07.2020 from: <https://www.facebook.com/AnnikenHolthe/posts/160355738771127>

Anniken Holthe Boxing (April 22nd 2020). In Facebook [Official athlete page]. Retrieved

29.07.2020 from: <https://www.facebook.com/AnnikenHolthe/posts/158448082295226>

Bergen Sør Bokseklubb (March 14^th^ 2020) In Facebook [Official club page]. Retrieved

30.07.2020 from: <https://www.facebook.com/bergenboxing365/posts/2936399326426853>

Bernard Angelo Torres (May 20th 2020). In Facebook [Official athlete page]. Retrieved

29.07.2020 from: <https://www.facebook.com/TeamTorresboxing/posts/945186315924882>

Bernard Angelo Torres (April 29th 2020). In Facebook [Official athlete page]. Retrieved

29.07.2020 from: <https://www.facebook.com/TeamTorresboxing/photos/a.311195435990643/929794157464098/?type=3>

Bernard Angelo Torres (April 15th 2020). In Facebook [Official athlete page]. Retrieved

29.07.2020 from: <https://www.facebook.com/TeamTorresboxing/posts/919424728501041>

Bernard Angelo Torres (March 26th 2020). In Facebook [Official athlete page]. Retrieved

29.07.2020 from: <https://www.facebook.com/TeamTorresboxing/posts/904454363331411>

Bernard Angelo Torres (March 21st 2020). In Facebook [Official athlete page]. Retrieved

29.07.2020 from: <https://www.facebook.com/TeamTorresboxing/posts/901136096996571>

Bernard Angelo Torres (March 19th 2020). In Facebook [Official athlete page]. Retrieved

29.07.2020 from: <https://www.facebook.com/TeamTorresboxing/posts/899773343799513>

Bodø Bokseklubb (May 29th, 2020). In Facebook [Official club page]. Retrieved 29.07.2020

from: <https://www.facebook.com/events/205787680389568/>

Bodø Bokseklubb (May 14th, 2020). In Facebook [Official club page]. Retrieved 29.07.2020

From: <https://www.facebook.com/events/313116986343674/>

Bodø Bokseklubb (May 12th, 2020). In Facebook [Official club page]. Retrieved 29.07.2020

From: <https://www.facebook.com/events/761487571052644/>

Bodø Bokseklubb (May 10th, 2020). In Facebook [Official club page]. Retrieved 29.07.2020

From: <https://www.facebook.com/events/252177169267109/>

Bodø Bokseklubb (May 7th, 2020). In Facebook [Official club page]. Retrieved 29.07.2020

From: <https://www.facebook.com/events/1594484954047828/>

Bodø Bokseklubb (May 4th, 2020). In Facebook [Official club page]. Retrieved 29.07.2020

From: <https://www.facebook.com/events/3025337287559279/>

Bodø Bokseklubb (April 30th, 2020). In Facebook [Official club page]. Retrieved 29.07.2020

From: <https://www.facebook.com/events/295459314774971/>

Bodø Bokseklubb (April 28th, 2020). In Facebook [Official club page]. Retrieved 29.07.2020

From: <https://www.facebook.com/events/171919357316697/>

Bodø Bokseklubb (April 24th, 2020). In Facebook [Official club page]. Retrieved 29.07.2020

From: <https://www.facebook.com/events/669034577251960/>

Bodø Bokseklubb (April 21st, 2020). In Facebook [Official club page]. Retrieved 29.07.2020

From: <https://www.facebook.com/events/154230942683998/>

Bodø Bokseklubb (April 19th, 2020). In Facebook [Official club page]. Retrieved 29.07.2020

From: <https://www.facebook.com/events/2814287882022824/>

Bodø Bokseklubb (April 15th, 2020). In Facebook [Official club page]. Retrieved 29.07.2020

From: <https://www.facebook.com/events/637592203471243/>

Bodø Bokseklubb (April 13th, 2020). In Facebook [Official club page]. Retrieved 29.07.2020

From: <https://www.facebook.com/events/2534778216838029/>

Bodø Bokseklubb (April 11th, 2020). In Facebook [Official club page]. Retrieved 29.07.2020

From: <https://www.facebook.com/events/156007489051411/>

Camilla Johansen (March28th 2020). In Facebook [Official athlete page]. Retrieved

29.07.2020 from: <https://www.facebook.com/camzijo/posts/2807011942713877>

Drammen Bokseklubb (March 12th 2020). In Facebook [Official club page]. Retrieved

30.07.2020 from: <https://www.facebook.com/DrammenKampsportsenter/posts/2956010877778810>

Horten Sportsklubb (March 27^th^ 2020). In Facebook [Official club page]. Retrieved

30.07.2020 from: <https://www.facebook.com/HortenSportsklubb/posts/866070347201405>

IL ROS Boksing (April 9^th^ 2020). In Facebook [Official club page] Retrieved 29.07.2020

from: <https://www.facebook.com/permalink.php?story_fbid=3227097944013255&id=469967096393034>

Jamshid Nazari (June 3d 2020). In Facebook [Official athlete page]. Retrieved 30.07.2020

from: <https://www.facebook.com/TeamNazari/photos/a.1223076864375814/3639408292742647/?type=3>

Jamshid Nazari (May 11th 2020). In Facebook [Official athlete page]. Retrieved 30.07.2020

from: <https://www.facebook.com/TeamNazari/photos/a.1223076864375814/3575940785756065/?type=3>

Jamshid Nazari (April 23d 2020). In Facebook [Official athlete page]. Retrieved 30.07.2020

from: <https://www.facebook.com/TeamNazari/posts/3528395930510551>

Jessheim Bokseklubb (April 3d, 2020). In Facebook [Official club page]. Retrieved

29.07.2020 from: <https://www.facebook.com/JessheimBokseklubb/posts/2643674459196963>

Jessheim Bokseklubb (April 4th, 2020). In Facebook [Official club page]. Retrieved

29.07.2020 from: <https://www.facebook.com/JessheimBokseklubb/posts/2644527969111612>

Jessheim Bokseklubb (April 5th, 2020). In Facebook [Official club page]. Retrieved

29.07.2020 from: <https://www.facebook.com/JessheimBokseklubb/posts/2645379865693089>

Jessheim Bokseklubb (April 6th, 2020). In Facebook [Official club page]. Retrieved

29.07.2020 from: <https://www.facebook.com/JessheimBokseklubb/posts/2646248242272918>

Jessheim Bokseklubb (April 8th, 2020). In Facebook [Official club page]. Retrieved

29.07.2020 from: <https://www.facebook.com/JessheimBokseklubb/posts/2647848565446219>

Jessheim Bokseklubb (April 17th, 2020). In Facebook [Official club page]. Retrieved

29.07.2020 from: <https://www.facebook.com/JessheimBokseklubb/posts/2655727754658300>

Jessheim Bokseklubb (May 2nd, 2020). In Facebook [Official club page]. Retrieved

29.07.2020 from: <https://www.facebook.com/JessheimBokseklubb/posts/2668835513347524>

King DON (April 4th 2020). In Facebook [Official athlete page]. Retrieved 29.07.2020 from:

<https://www.facebook.com/2057925467602970/photos/a.2060195367375980/2990501814345326/?type=3>

King DON (March 28th 2020). In Facebook [Official athlete page]. Retrieved 29.07.2020

from: <https://www.facebook.com/permalink.php?story_fbid=2959047314157443&id=2057925467602970>

King DON (March 19th 2020). In Facebook [Official athlete page]. Retrieved 29.07.2020

from: <https://www.facebook.com/permalink.php?story_fbid=2956908754371299&id=2057925467602970>

Madeleine Angelsen (n.d., 2020). Blog [Official athlete blog]. Retrieved 29.07.2020 from:

<http://www.madeleineangelsen.com/?fbclid=IwAR2NoyYxE1yA8I29hNYMd_XSq9MJ6CR06rNusPKVz02T_tNnjYec36P0nUA>

Mindaugas Gedminas (May 9^th^ 2020). In Facebook [Official athlete page]. Retrieved

29.07.2020 from: <https://www.facebook.com/Gedminas.boxing/posts/789390534927483>

Moldekameratene Bokseklubb (May 11^th^ 2020). In Facebook [Official club page]. Retrieved

29.07.2020 from: <https://www.facebook.com/moldekameratene/posts/2570780296475913>

Moldekameratene Bokseklubb (April 16^th^ 2020). In Facebook [Official club page]. Retrieved

29.07.2020 from: <https://www.facebook.com/moldekameratene/posts/2550957341791542>

Moldekameratene Bokseklubb (March 19^th^ 2020). In Facebook [Official club page]. Retrieved

29.07.2020 from: <https://www.facebook.com/moldekameratene/posts/2527852790768664>

Moldekameratene Bokseklubb (March 16^th^ 2020). In Facebook [Official club page]. Retrieved

29.07.2020 from: <https://www.facebook.com/moldekameratene/posts/2525705284316748>

Oslo Bokseklubb (June 1^st^ 2020). In Facebook [Official club page]. Retrieved 29.07.2020

from: <https://www.facebook.com/OsloBokseklubb/posts/1639500019540574>

Oslo Bokseklubb (May 19th 2020). In Facebook [Official club page]. Retrieved 29.07.2020

from: <https://www.facebook.com/OsloBokseklubb/posts/1627511227406120>

Oslo Bokseklubb (May 14th 2020). In Facebook [Official club page]. Retrieved 29.07.2020

From: <https://www.facebook.com/OsloBokseklubb/posts/1623597131130863>

Simen Nysæter (April 4^th^  2020 ). In Facebook [Official athlete page]. Retrieved 29.07.2020

from: <https://www.facebook.com/simennysaeter/posts/2778440572377169>

Raufoss Bokseklubb (March 16^th^ 2020). In Facebook [Official club page]. Retrieved

30.07.2020 from: <https://www.facebook.com/kyrrekvist/posts/1588418134640279>

Romerike Bokseklubb (March 28^th^ 2020). In Facebook [Official club page]. Retrieved

30.07.2020 from: <https://www.facebook.com/romerikebokseklubb/posts/2534995823483931>

Romerike Bokseklubb (March 23^d^ 2020). In Facebook [Official club page]. Retrieved

30.07.2020 from: <https://www.facebook.com/romerikebokseklubb/posts/2533161773667336>

Romerike Bokseklubb (March 18^th^ 2020). In Facebook [Official club page]. Retrieved

30.07.2020 from: <https://www.facebook.com/romerikebokseklubb/posts/2528531147463732>

Romerike Bokseklubb (March 16^th^ 2020). In Facebook [Official club page]. Retrieved

30.07.2020 from: <https://www.facebook.com/romerikebokseklubb/posts/2526484904335023>

Romerike Bokseklubb (March 14^th^ 2020). In Facebook [Official club page]. Retrieved

30.07.2020 from: <https://www.facebook.com/romerikebokseklubb/posts/2524632674520246>

Skien Bokseklubb (May 11^th^ 2020). In Facebook [Official club page]. Retrieved 30.07.2020

from: <https://www.facebook.com/SkienBokseklubb/posts/2024773814314614>

Skien Bokseklubb (March 14^th^ 2020). In Facebook [Official club page]. Retrieved 30.07.2020

from: <https://www.facebook.com/SkienBokseklubb/posts/1912226208902709>

TeamTorresChannel (March 25^th^ 2020). In YouTube [Official athlete page]. Retrieved

29.07.2020 from: <https://www.youtube.com/watch?v=xzAFNrKK5BU&t=2s&fbclid=IwAR1J98PeG-2nuOSVi3uFmvs3DevXeLmooXm4PhMPt91eibOpITs6YxvbsIw&app=desktop>

TeamTorresChannel (March 22^nd^ 2020). In YouTube [Official athlete page]. Retrieved

29.07.2020 from: <https://www.youtube.com/watch?v=mWgmZ32pyQg>

The Norwegian Combat Academy (April 23d, 2020). In Facebook [Official club page].

Retrieved 29.07.2020 from: <https://www.facebook.com/thenorwegiancombatacademy/posts/650952912133970>

The Norwegian Combat Academy (April 20th, 2020). In Facebook [Official club page].

Retrieved 29.07.2020 from: <https://www.facebook.com/thenorwegiancombatacademy/posts/649123582316903>

The Norwegian Combat Academy (April 11th, 2020). In Facebook [Official club page].

Retrieved 29.07.2020 from: <https://www.facebook.com/thenorwegiancombatacademy/posts/643194162909845>

The Norwegian Combat Academy (April 10th, 2020). In Facebook [Official club page].

Retrieved 29.07.2020 from: <https://www.facebook.com/thenorwegiancombatacademy/posts/642565666306028>

The Norwegian Combat Academy (April 8th, 2020). In Facebook [Official club page].

Retrieved 29.07.2020 from: <https://www.facebook.com/thenorwegiancombatacademy/posts/641370053092256>

The Norwegian Combat Academy (April 6th, 2020). In Facebook [Official club page].

Retrieved 29.07.2020 from: <https://www.facebook.com/thenorwegiancombatacademy/posts/640115503217711>

The Norwegian Combat Academy (April 3d, 2020). In Facebook [Official club page].

Retrieved 29.07.2020 from: <https://www.facebook.com/thenorwegiancombatacademy/posts/638256723403589>

The Norwegian Combat Academy (April 1st, 2020). In Facebook [Official club page].

Retrieved 29.07.2020 from: <https://www.facebook.com/thenorwegiancombatacademy/posts/637173153511946>

The Norwegian Combat Academy (March 30th, 2020). In Facebook [Official club page].

Retrieved 29.07.2020 from: <https://www.facebook.com/thenorwegiancombatacademy/posts/635991320296796>

The Norwegian Combat Academy (March 27th, 2020). In Facebook [Official club page].

Retrieved 29.07.2020 from: <https://www.facebook.com/thenorwegiancombatacademy/posts/634031670492761>

The Norwegian Combat Academy (March 26th, 2020). In Facebook [Official club page].

Retrieved 29.07.2020 from: <https://www.facebook.com/thenorwegiancombatacademy/posts/633512763877985>

The Norwegian Combat Academy (March 25th, 2020). In Facebook [Official club page].

Retrieved 29.07.2020 from: <https://www.facebook.com/thenorwegiancombatacademy/posts/632618000634128>

TK Tønsbergkameratene Boxing club (March 12th 2020). In Facebook [Official club page].

Retrieved 30.07.2020 from: <https://www.facebook.com/tkboksing/posts/2916618338381047>
